# Reaction Monitoring and Structural Characterisation of Coordination Driven Self-Assembled Systems by Ion Mobility-Mass Spectrometry

**DOI:** 10.3389/fchem.2021.682743

**Published:** 2021-06-08

**Authors:** Oscar H. Lloyd Williams, Nicole J. Rijs

**Affiliations:** School of Chemistry, UNSW Sydney, Sydney, NSW, Australia

**Keywords:** self-sorting, supramolecular structure, aggregation, dynamic combinatorial library, complex solutions, topology, coordination polymer, structural dynamics

## Abstract

Nature creates exquisite molecular assemblies, required for the molecular-level functions of life, via self-assembly. Understanding and harnessing these complex processes presents an immense opportunity for the design and fabrication of advanced functional materials. However, the significant industrial potential of self-assembly to fabricate highly functional materials is hampered by a lack of knowledge of critical reaction intermediates, mechanisms, and kinetics. As we move beyond the covalent synthetic regime, into the domain of non-covalent interactions occupied by self-assembly, harnessing and embracing complexity is a must, and non-targeted analyses of dynamic systems are becoming increasingly important. Coordination driven self-assembly is an important subtype of self-assembly that presents several wicked analytical challenges. These challenges are “wicked” due the very complexity desired confounding the analysis of products, intermediates, and pathways, therefore limiting reaction optimisation, tuning, and ultimately, utility. Ion Mobility-Mass Spectrometry solves many of the most challenging analytical problems in separating and analysing the structure of both simple and complex species formed via coordination driven self-assembly. Thus, due to the emerging importance of ion mobility mass spectrometry as an analytical technique tackling complex systems, this review highlights exciting recent applications. These include equilibrium monitoring, structural and dynamic analysis of previously analytically inaccessible complex interlinked structures and the process of self-sorting. The vast and largely untapped potential of ion mobility mass spectrometry to coordination driven self-assembly is yet to be fully realised. Therefore, we also propose where current analytical approaches can be built upon to allow for greater insight into the complexity and structural dynamics involved in self-assembly.

## Introduction

### Do Complex Systems Require Complex Analyses?

As chemists, how do we get a handle on dynamic systems? One approach is to simplify and study model systems. But if we could “simplify” the weather, would we be able to accurately predict a cyclone? Or a once-in-a-century drought? Or even the difference in average rainfall between the seasons? No. Complex systems lead to complex behaviours and different-than-the-sum-of-the-average endpoints, and over simplifying will not most often lead to correct predictions of interesting or “emergent” behaviour, such as non-thermodynamic outcomes (Ashkenasy et al., 2017). Our understanding of molecular-level structure-function relationships has facilitated the rational design of compounds and materials for highly specific functions. For example, zeolites have highly engineered catalytic properties based on pore and active site structure (Vjunov et al., 2014), while sophisticated supramolecular coordination cages have been recently developed as reaction vessels, promoting a range of encapsulated chemoselective, and even enantioselective reactions (Brown et al., 2015; Liu and Stoddart, 2021). A corollary of this functionality, however, is that as the desired molecular complexity increases, so too does the synthetic challenge of preparing the desired structures in an energy- and atom-efficient manner; amenable to industrial production. So too, does the analytical challenge increase, as the complexity of pathways, intermediates, products and “off-cycle” side-products increases, confounding detection of one another and jostling for our finite attention. Does our analytical toolkit need expanding as we begin to embrace this complexity? In this review we highlight ion mobility mass spectrometry as an elegant solution to many of the “wicked” analytical challenges presented when embracing such complexity, particularly that generated via coordination driven self-assembly.

### A Window or a Snapshot: Optimising Self-Assembly

Taking inspiration from nature, functional materials can be constructed via self-assembly (SA), where a thermodynamically downhill route generates molecular complexity and imbues the desired utility. Preferably, processes should occur either spontaneously, or with minimum intervention, and occur on a practical timescale. Building blocks that utilise metal-ligand coordination chemistry to generate predefined structure are promising artificial SA systems (Leininger et al., 2000; Linder et al., 2015). Metal-ligand SA is being utilized to generate a startling array of bio-inspired complexes with intriguing properties, for example artificial systems which undergo cell-like chemical cascades in response to stimuli (Campbell et al., 2010; He et al., 2015). As the structures of self-assembled materials are often wide-ranging, so too are their formation mechanisms, which are not yet as widely understood as other types of chemical transformations (e.g., synthetic organic reactions and catalysis). While the thermodynamic end point in SA is often aimed for and characterised, intermediate structures and metastable states are typically unknown; being largely analytically inaccessible. Yet, the rate and overall selectivity of the SA process are not only thermodynamically driven but are dependent on these intermediate and other transitory states (e.g., via self-organisation). By analogy with catalytic “off-cycle” pathways, undefined “off-pathway” reaction processes occurring during SA inevitably lead to a reduction in overall efficiency (Aliprandi et al., 2016). As pointed out by Miras *et al.*, *“…self-assembly processes are highly dependent upon the reaction conditions, often to such a degree that total control is never easily achieved*” (Miras et al., 2009). Thus, despite the selectivity of SA, its industrial usefulness is currently limited due to an inability to systematically enhance its efficiency. Recent landmark work showed that solvent composition and light can be judiciously employed to affect the pathways of SA—a paradigm shifting result—suggesting that synthetic targets outside the range of thermodynamic minima are possible (Aliprandi et al., 2016). Being able to monitor the reaction intermediates and kinetics in solution, in order to understand and control reactivity and selectivity, is critical to optimising these chemistries. While optical spectra (e.g., UV-visible absorption spectra) can be employed to monitor the reactions (Misuraca et al., 2014), not all assemblies possess the necessary photo-physical properties. Furthermore, optical spectroscopy often lacks the structural resolution to unambiguously assign supramolecular connectivity. These challenges are compounded by the presence of isomeric intermediates that share a common stoichiometry, but have distinct connectivity. The lack of analytical methods to intercept the intermediates in SA reactions is a major impediment to developing robust models and the rational optimisation of the process. In the same way, there exists an absence of effective analytical probes for assessing the structure-function relationships of complex molecular assemblies. The function of molecular assemblies—such as the inclusion of molecules within a capsule, are inherently difficult to study in isolation and therefore are often poorly defined, limiting their application or further rational optimization. Indeed, by all accounts this is the frontier, as Whitesides has identified “*…the most important problems for analytical chemistry may be changing from “molecules” to “functions”* (Whitesides, 2013).

## Self-Assembly and Coordination Driven Self-Assembly

**FIGURE 1:**
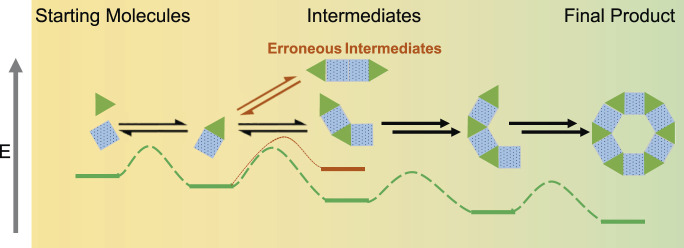
Simplified example of the self-assembly of Starting “building block” Molecules into Final “assembled” Product through repeated association and dissociation events that produce intermediates, erroneous intermediates, and side products. Note that each step of self-assembly is reversible, and therefore the entire process is in dynamic equilibrium (Whitelam and Jack, 2015).

Self-assembly processes occurring under ambient conditions are generally considered to be under thermodynamic control. In this scenario, a thermodynamic end product is eventually formed through repeat association and dissociation events (Figure 1) (Whitesides and Boncheva, 2002). Chemists may design new systems simply by modifying the starting “building block” molecules and observing the final “assembled” product. So called “erroneous” intermediates may also associate and dissociate competitively in “off-cycle” pathways. The ratio of intermediates and product shifts towards products with time. To apply a rational design process, as opposed to a serendipitous one, an understanding of the structure-activity relationships that govern self-assembly within a given equilibrating system is required. In turn this allows targeted properties in the final assembled products to be achieved efficiently. Additionally, if non-equilibrium products are the desired target, an approach to shift and optimise the pathways can only be attempted with this understanding.

Self-assembly is usually categorised by the dominant non-covalent interactions that govern the process, such as hydrogen bonding, hydrophobic interactions, π-π interactions or metal-ligand coordination (Whitesides and Boncheva, 2002). Coordination driven self-assembly (CDSA) uses the formation of coordination bonds as the driver for self-assembly. The most common are the coordination bonds between Lewis acid metal centres and Lewis base ligands, but other coordination systems exist, notably anion coordination (Yang et al., 2018). One example that highlights the versatility of coordination chemistry is the comprehensive work performed by Link *et al.* with *m*-xylene macrocycles (Link et al., 2020). These analytes were found to coordinate with cations and anions to self-assemble into oligomers.

It is worth noting that whilst a coordination bond is, electronically, a dative covalent bond and is stronger than most other non-covalent interactions (ca. 100 kJ mol^−1^), it is still far more labile than a standard covalent bond (ca. 400 kJ mol^−1^), allowing it to thermodynamically equilibrate under milder conditions (Table 1). Highly functional materials have been synthesized with CDSA, the wide diversity of utility including complexes with, for example, antimicrobial properties (Wang et al., 2019), that enhance catalysis (Kuijpers et al., 2016; Fulong et al., 2019), and that are used as drug delivery systems (Casini et al., 2017; Fulong et al., 2019; Wang et al., 2019).

**TABLE 1:** Table of example relative bond strengths, highlighting the intermediate nature of metal ligand dative covalent bonds.

Bond type	Bond	Approx. bond energy/kJ mol^−1^
Hydrogen	OH-:O (in water)	23^a^
Metal ligand (dative)	Zn-O	180^b^
Metal ligand (dative)	Cu-N	90^b^
Covalent	C-C	376^c^
Covalent	C-H	438^c^

a The hydrogen bond energy was obtained from the application of statistical mechanical principles and the known dielectric constant of water and matches closely with experimental data (Suresh and Naik, 2000).

b The metal-ligand values are derived from work performed analysing the Cambridge Structural Database. Several different crystal structures were investigated to obtain estimates for metal ligand bond strengths (Anders et al., 2013).

c The organic covalent bond strengths were derived experimentally by three different experimental techniques (radical kinetics, photoionization mass spectrometry, acidity/electron affinity cycle) (Blanksby and Ellison, 2003).

Owing to the increased strength of the coordination bonds relative to other intermolecular bonds (c.f. Hydrogen bonding, which is ca. 20 kJ mol^−1^ and an order of magnitude weaker than dative bonding), coordination based supramolecular structures are relatively stable compared to other non-covalent structures. However, this strength also leads to longer self-assembly timescales, as erroneous assemblies can become “kinetically trapped” by large energy barriers between mis-bound and unbound states, due to the stability conferred by the coordination bonds (Whitelam and Jack, 2015). The directionality of metal-ligand covalent bonds can also be exploited with well-designed multidentate ligands to create metal ligand systems that readily self-assemble into either discrete polygons and polyhedra, or for example, continuous coordination polymers (Hoskins and Robson, 1989; Northrop et al., 2008).

### Analytical Challenges of Monitoring and Characterising Products and Intermediates of Self-Assembly

Traditional small molecule techniques such as NMR, TGA, UV-Vis spectroscopy, X-ray crystallography and mass spectrometry have all been successfully applied to understanding the mechanisms of coordination driven self-assembly, in the solid, liquid and gas phases (Table 2) (Liao et al., 2018; Singh et al., 2020). Methods such as X-ray crystallography and TEM rely on isolating final products, limiting their ability to study the dynamic nature of self-assembly (Liao et al., 2018). Other techniques, such as NMR, UV-Vis, and mass spectrometry, allow for the monitoring and probing of structural dynamics, over various time scales (Schalley, 2007).

**TABLE 2:** Techniques and their analytical strengths and weaknesses in the context of analysing CDSA structures and intermediates.

Technique	Sample type	Strength	Weakness
NMR	Liquid, solid	Can measure intermolecular environments	Relies on presence of spin active nuclei
		Can be applied dynamically	Overlapping signals present assignment challenges
		Certain methods, e.g NOESY, can provide intermolecular structural information	Difficulties with radicals
DOSY NMR	Liquid, solution	Excellent for determining size of molecules within mixtures.	Relies on presence of spin active nuclei
		Can be layered over other NMR experiments.	Can struggle with larger molecules that diffuse slowly Can be affected by exchange processes
DLS	Solution or suspension	Can accurately measure the size and shape of macromolecules and supramolecules	Struggles to discern structurally similar molecules (monomers, dimers)
		Can study interactions between analytes	Structures formed in short timescale obscured by larger ones
TEM	Ultrathin solid	Allows direct structural characterisation	Samples must be “electron transparent”
			Sample preparation can be challenging
			Not dynamic
TGA	Solid or liquid	Can give insight into the thermodynamic driving forces of self- assembly	Structural information obtained is limited
UV-Vis spectroscopy	Solution	Effective for measuring transition elements and conjugated organic molecules	Limited available structural information
		Characteristic absorbances reveal (supra)molecular changes	All molecules in solution can give signal, leading to overlapping signals
		Fast measurement allows dynamic measurements and reaction monitoring	Target species must be spectroscopically active
ITC	Solution	Quantitative measure of binding affinities and thermodynamics	Measures equilibrium conditions, not suited to non-equilibrium systems
			Cannot distinguish individual components from less specific contributions of bulk solution
X-ray Crystallography	Crystalline solid	Can obtain full structure, including isomerism	Dependant on successful crystallisation
		Self-assembled structures can be preserved in crystal	Does not capture dynamic process
		Information about bonding	
ESI-MS or MALDI-MS	Solid, liquid, gas	Empirical formulas and charge states easily distinguished	Target species must be ionisable/carry a charge
		Structural information through MS^n^	Artifacts due to ionisation process
		Time scale dynamic range (milliseconds – hours) allows for dynamics and reaction monitoring	Cannot decern all isomers
		Dynamic range of masses allows simultaneous monitoring of reactant and product	Isobaric interferences
		Fragile species can be detected	
		Can be automated, high throughput	
IMS	Solid, liquid, gas	In addition to advantages for MS, can filter isomers and isobars	Target species must be ionisable/carry a charge
		Structural identification via collisional cross section (Ω or CCS)	Fragile species can be fragmented

The primary challenge for many techniques that do have the ability to study dynamics is differentiating the signals from individual components and the signals from the same components incorporated within different supramolecular structures. Dynamic light scattering (DLS) approaches allow the structural dynamics to be studied with temporal resolution, but small units formed at short timescales are masked by the formation of larger units (Jouault et al., 2015). NMR can often differentiate between the supramolecule and its component small molecules. However mixtures of assemblies formed from the same small molecules will often be impossible to accurately analyse with NMR techniques, as unique chemical shifts will not be obtained for each structure present (Endres et al., 2019). This limits some of the applications of NMR. Diffusion Ordered NMR Spectroscopy (DOSY) has been employed as it can separate molecules and report the diffusion coefficients of analytes, a property linked to the size of an assembly, but it has been reported in some cases that assemblies of the same size will not diffuse on the NMR timescale (Ebbert et al., 2019). This again leads to mixtures of signals that cannot be used to unambiguously assign structures (Misuraca et al., 2014). While diamagnetic metals are readily characterized, NMR techniques are also typically less useful for paramagnetic metals and radical ligands. Additionally, as ^1^H NMR is often employed, it is simpler to characterize symmetrical assemblies, leading to biases which discourage interpretation, and even initial synthetic targeting, of heteroleptic mixtures.

**FIGURE 2:**
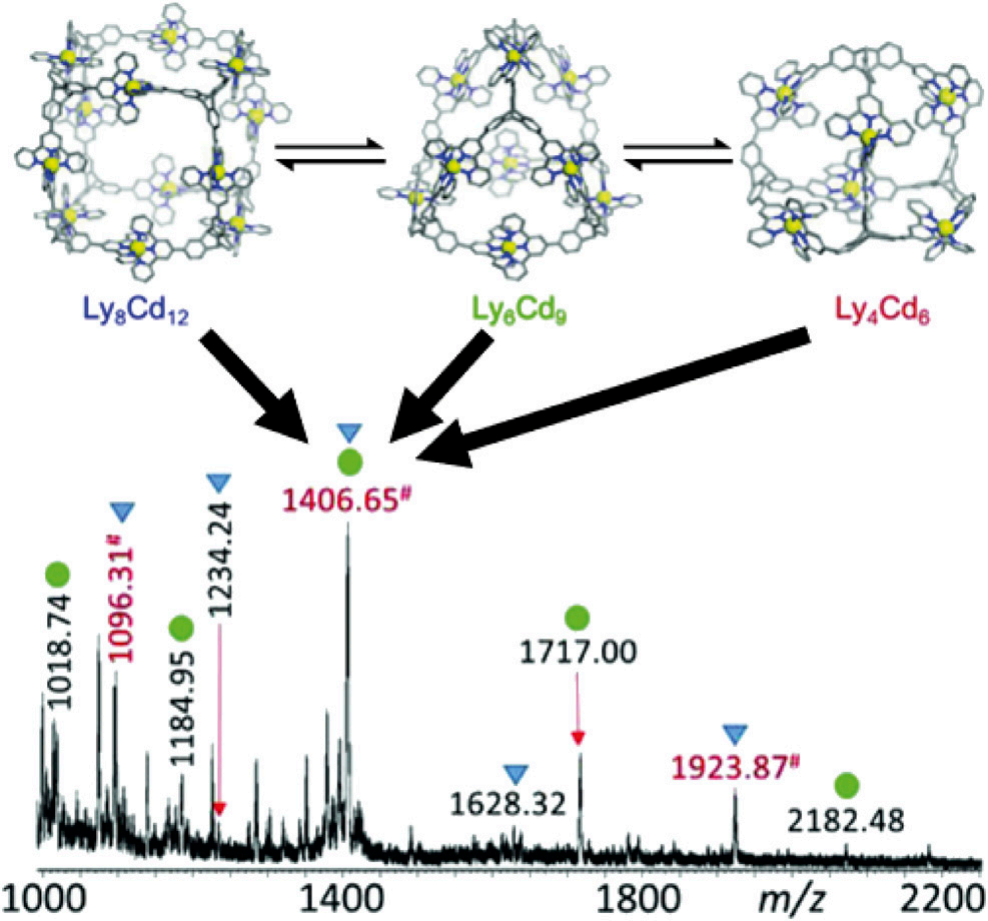
An example of isobaric species occluding each other; the peak at *m/z* 1,406.65 is a combination of octomer, hexamer and tetramer structures of the same tris-terpyridine based ligand and cadmium (Endres et al., 2019).

In mass spectrometry, whilst molecular components and supramolecular assemblies are easily distinguishable, species sharing the same nominal mass or *m/z* ratio, but different molecular formula (including isotopologues), termed “isobars,” are not. This applies in the case of hierarchal supramolecules. For example, analysis of metallo-macromolecular assemblies made up of the same subunits with electrospray ionisation mass spectrometry (ESI-MS) reveal that the 4^+^ tetramer and a 6^+^ hexamer, which share the same nominal *m/z* ratio, occlude each other, hindering analysis by multistage mass spectrometry (MS/MS) (Figure 2) (Endres et al., 2019). Indeed, even the molecular subunits themselves can be isomers through processes such as ligand isomerisation (Rijs et al., 2014), often escaping scrutiny due to the challenge of identification within the complex mixtures.

**FIGURE 3:**
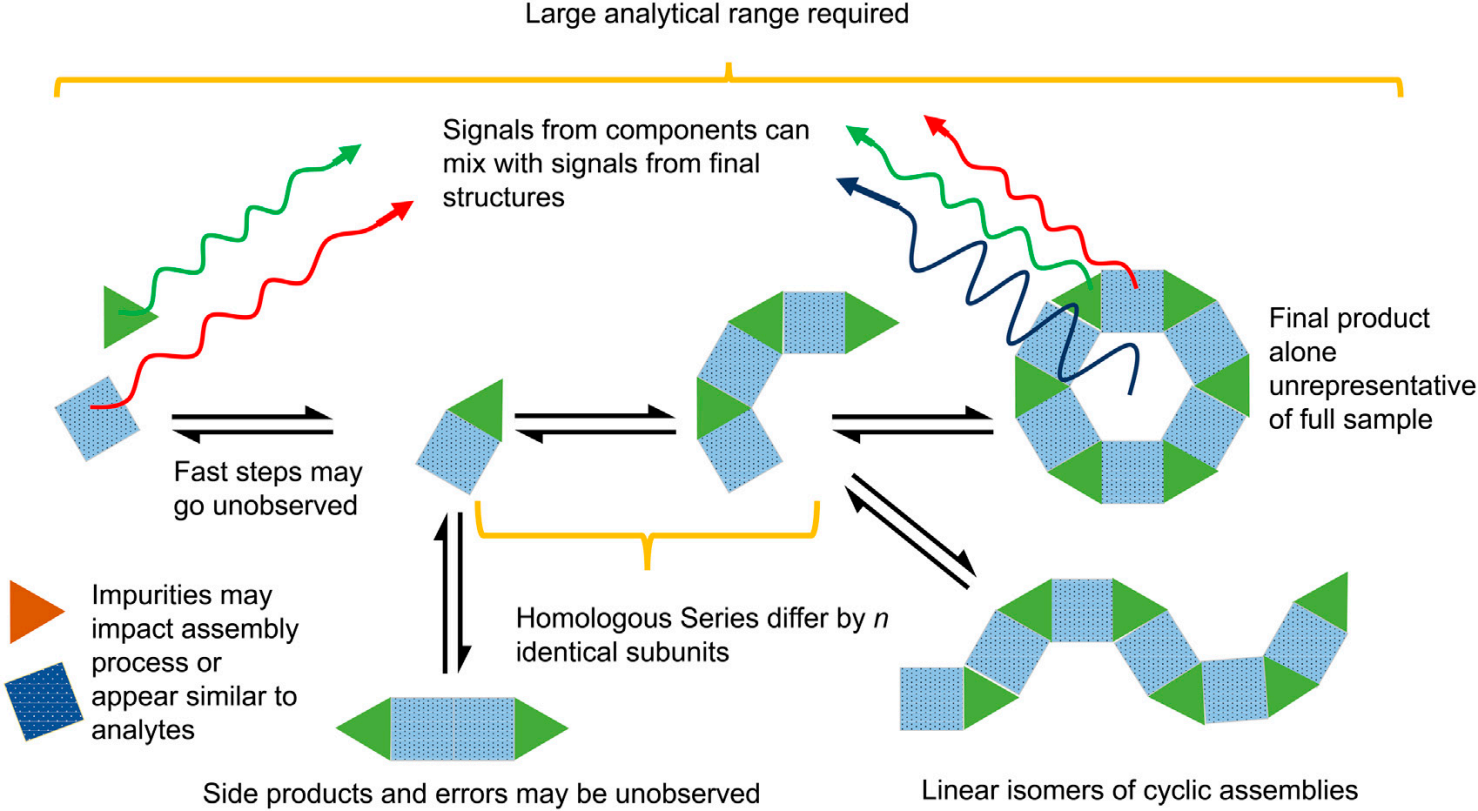
The not-insignificant challenges that must be overcome when analysing self-assembly. These include the large analytical range, mixed signals, homologous series, and variable timescales.

The challenge of identifying and resolving occluded species is non-trivial to the study of dynamic libraries of self-assembling species in solution (Figure 3) (Misuraca et al., 2014). Mass spectrometry gives ready access to real time analysis of dynamic systems on a millisecond timescale (Schalley and Springer, 2009; Hudgens et al., 2011; Pettibone and Hudgens, 2012; Olivares et al., 2014; Yunker et al., 2014; Ligare et al., 2017a; Ligare et al., 2017b; Ujma et al., 2017; Schnell et al., 2019; Mehara and Roithová, 2020; Zelenka and Roithová, 2020). The difficulties of occluded isobaric and isomeric species can be overcome by adding a filter for shape and size, in addition to *m/z*, namely, an ion-mobility spectrometer (IMS) (Ujma et al., 2017; Polewski et al., 2021). Further, accurate determination of supramolecular structures and their constituent components, simultaneously, can be made (Kalenius et al., 2019).

## Ion Mobility-Mass Spectrometry

### Ion Mobility-Mass Spectrometry Principles

Ion mobility spectrometry separates ions by their gas phase mobility in an electric field, a property which is linked to their three-dimensional structure (viz., shape and topology) and charge. Mass spectrometry uses electromagnetic fields to separate ions by their mass-to-charge ratios (*m/z*). Ion mobility mass spectrometry (IMS-MS) is thus the hyphenation of the two gas phase techniques (Cumeras et al., 2015b). Whilst not fully orthogonal (mass-to-charge and shape-to-charge being related to a certain degree), their hyphenation yields another complementary dimension (IMS) to the already information rich MS data. This provides a satisfying solution to the challenge of separating and identifying isobaric and isomeric species. IMS-MS is now well-regarded as an analytical technique and is perfectly suited to interrogating self-assembly.

The mobility of an ion is described by its mobility coefficient (*K*), the velocity per unit electric field (Eq. 1). The mobility coefficient can be linked to the collision cross section and charge of the ion through Eq. 2 (Smith et al., 2009). The collision cross section (Ω) of an ion is the “orientationally averaged collision integral,” (Lee et al., 2017), which describes the area of space that a rotationally averaged ion occupies that would scatter a gas molecule, a value directly linked to structure (Marchand et al., 2017). This value not only contains the area where a gas molecule could collide with the molecule directly, but also the area surrounding the molecule where electrostatic interactions could occur and scatter a gas molecule, despite no direct collision. It is noteworthy that this value is relatively independent of the nature of the bonds within the structures observed. This lends it a flexibility in investigating self-assembled complexes and supramolecular mixtures possessing several bond types, including weaker intermolecular interactions, in a non-targeted fashion.

$$K=\frac{v_{d}}{E}$$ (1)

Equation 1: In a simple case the mobility coefficient of a given ion, *K* (m^2^ V^−1^ s^−1^), is the ratio of the ionic drift velocity, *V*_*d*_ (m s^−1^) to the electric field strength *E* (v m^−1^).

$$K=\frac{3ze}{16N}{\left(\frac{2\pi }{\mu k_{B}T}\right)}^{\left(1/2\right)}\frac{1}{\Omega }$$ (2)

Equation 2: The Mason-Schamp equation describing the relationship between mobility coefficient (*K*) and collision cross section (Ω) (Revercomb and Mason, 1975), where *μ* is the reduced mass, *k*_*B*_ is the Boltzmann constant, *T* is the temperature of the ion, *z* is the whole number charge of the ion, *e* is the electron charge and *N* is the number volume of neutral gas molecules of the buffer gas.

### Ion Mobility-Mass Spectrometry Instrumentation

Though ion mobility spectrometers have been reviewed in depth (Borsdorf and Eiceman, 2006; Kanu et al., 2008; Wyttenbach et al., 2011; Cumeras et al., 2015a; May and McLean, 2015; Eiceman et al., 2016; Dodds and Baker, 2019; Kirk et al., 2019), here we provide an instrumentation overview in the context of CDSA analysis. There are many different ion mobility instrument configurations. These can be at high or low electric field (*E*) (Cumeras et al., 2015a). The benefit of low field ion mobility is that the relationship between *K* and Ω is determinable. Thus, ions can be separated according to their mobility coefficients, and the measured *K* used to derive experimental collisional cross sections, Ω_exp_. Mixtures of unknown structures are ubiquitous in CDSA systems. Low field IMS is therefore a powerful method for simultaneously separating and confirming the structure of ions observed. Three of the most common low field ion mobility spectrometers are Drift Tube Ion Mobility Spectrometers (DTIMS), Trapped Ion Mobility Spectrometers (TIMS) and Travelling Wave Ion Mobility Spectrometers (TWIMS).

**FIGURE 4:**
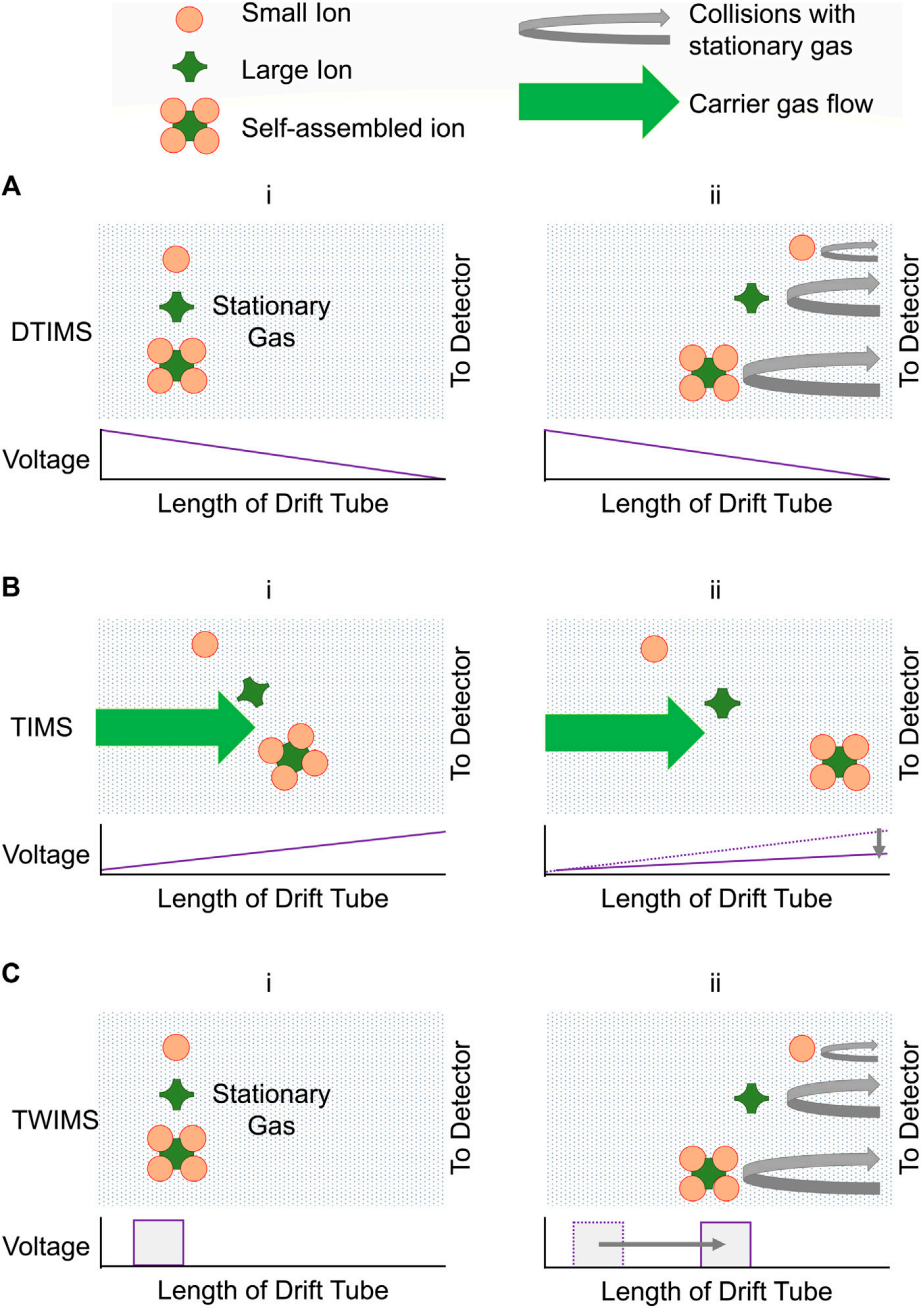
Several common ion mobility spectrometers that result in ions of different sizes/topologies being separated in time. The mode of action of: **(A)** Drift Tube Ion Mobility Spectrometry (DTIMS), **(i)** accelerates swarms of ions with an electric field and **(ii)** their kinetic energy is reduced by varying amounts by collision with stationary gas molecules; **(B)** Trapped Ion Mobility Spectrometry (TIMS), **(i)** separates swarms of relatively stationary ions through collision with moving drift gas and **(ii)** eluting the ions in turn by decreasing the trapping potential; **(C)** Travelling Wave Ion Mobility Spectrometry (TWIMS) **(i)** pushes swarms of ions against a stationary gas using travelling voltage waves to move the ions down the cell over time, some more efficiently than others. **(ii)** the ions become distributed according to their topologies as they are carried along with various efficiencies and kinetic energy is reduced by varying amounts by collision with stationary gas molecules.

DTIMS is conceptually simplest of the three, using a uniform electric field to propel ions in one direction, whilst a stationary drift gas separates ion by reducing the kinetic energy of ions with larger Ω values (Figure 4A) (Gabelica et al., 2019). The traversal time is, intuitively, proportional to the collision cross section, itself inversely proportional to mobility. A limiting factor in the use of DTIMS is that separation is proportional to the length of the drift tube, putting a practical limit on the achievable resolution. Heat may be used to increase separation.

TIMS is a relatively novel inversion of DTIMS, using an electric field to hold ions in a narrow area, whilst moving gas separates the ions within that area (Figure 4Bi) (Fernandez-Lima et al., 2011). Once the electric field is reduced, ions are “eluted” from the instrument in order of mobility (Figure 4Bii) and the mobility can easily be linked to Ω through Eq. 2 (Ridgeway et al., 2018). A benefit of TIMS is that it can accumulate ions for an extended period, allowing it to better function with long cycle time MS instruments and increase sensitivity.

TWIMS propels ions through a gas with pulsing voltage waves that travel the length of the device. As they do, ions with greater Ω values experience more collisions, increasing the time spent in the device. The potential waves themselves also carry some species more efficiently than others (Hines et al., 2016). Ions are separated by size, with larger ions travelling through the instrument more slowly (Figure 4C). The relationship between V_d_ and K is no longer described by Eq. 1, as the ions do not experience a constant electric field**,** but by more complex equations (Shvartsburg and Smith, 2008). Thus, to derive Ω_exp_, drift time data obtained from a TWIMS instrument are calibrated. Using calibrants of a known and well-defined Ω, the parametrised equation is solved through fitting, and is highly reproducible. The IMS calibrants should be a similar structural motif to the analyte, the same charge state, and all experiments carried out under identical instrumental conditions. An important consideration in all IMS techniques is that some species, even within the same structural motif, may potentially interact with the background nitrogen buffer gas more than others. This is particularly relevant to metal complexes studied with TWIMS (as TWIMS relies on nitrogen) and must be considered when comparing Ω values (Rijs et al., 2015a; Rijs et al., 2015b). However, note this effect can be highly beneficial to separation of isomers with a very small difference in overall Ω (Rijs et al., 2014; Rijs et al., 2016). There is a potential heating effect of the intense electric field of TWIMS, which may induce dissociation in smaller molecules (Firouzbakht et al., 2016; Firouzbakht et al., 2018), but in larger structures could induce rearrangement of structurally labile complexes, an effect that may prove critical when observing CDSA supramolecules (Merenbloom et al., 2012; Morsa et al., 2014). Nonetheless, strengths of TWIMS include IMS resolution over a short distance, tunability and ion transmission, which gives it a flexible analyte range and ability to be used in tandem separations and instrument configurations. The uptake of TWIMS for studying CDSA has been marked.

Beyond the comparison and reporting of Ω values, reporting reduced mobility values (*K*_0_) is a recommended practice that could provide a unique identifying value linked to the structure of the analyte (Gabelica et al., 2019). These values are important because they are specific to the molecule in question, whilst being independent of the ion mobility technique used. This is achieved by normalising the mobility value with respect to a standard temperature (273.3 K) and pressure (760 torr) as shown in Eq. 3 (Smith et al., 2009). As such an accurate *K*_0_ could be used as an identification tool across multiple instruments, and implementation of databases (May and McLean, 2015). For accurate determination of *K*_0_, the temperature and pressure of the buffer gas should be accurately determined.

In high field or field asymmetric ion mobility (FAIMS) techniques, ions experience a varied electric field at any given moment and the mobility of an ion is dependant on field strength (Kolakowski and Mester, 2007). As such obtaining Ω values is not as straight forward as for low field techniques. These techniques can enhance separation, signal to noise, and resolve targeted isobars, but they are less helpful for characterization of species within complex mixtures. FAIMS has been demonstrated to isolate and enhance the transmission of individual ions from within an isobaric mixture of self-assemblies (Arthur et al., 2016). However, the focus here is the application of low field techniques.

$$K_{0}=K\frac{273.2}{T_{\text{Buffer}\;\text{Gas}}}\frac{P_{\text{Buffer}\;\text{Gas}}}{760}$$ (3)

Equation 3: Reduced mobility, *K*_0_, is readily calculated from an experimental mobility using temperature and pressure values for the buffer gas used in the experiment. The values used are normalising the value to STP (273.2 K, 760 Torr).

### Methods for Predicting Candidate Structures for Ion Mobility-Mass Spectrometry

**FIGURE 5:**
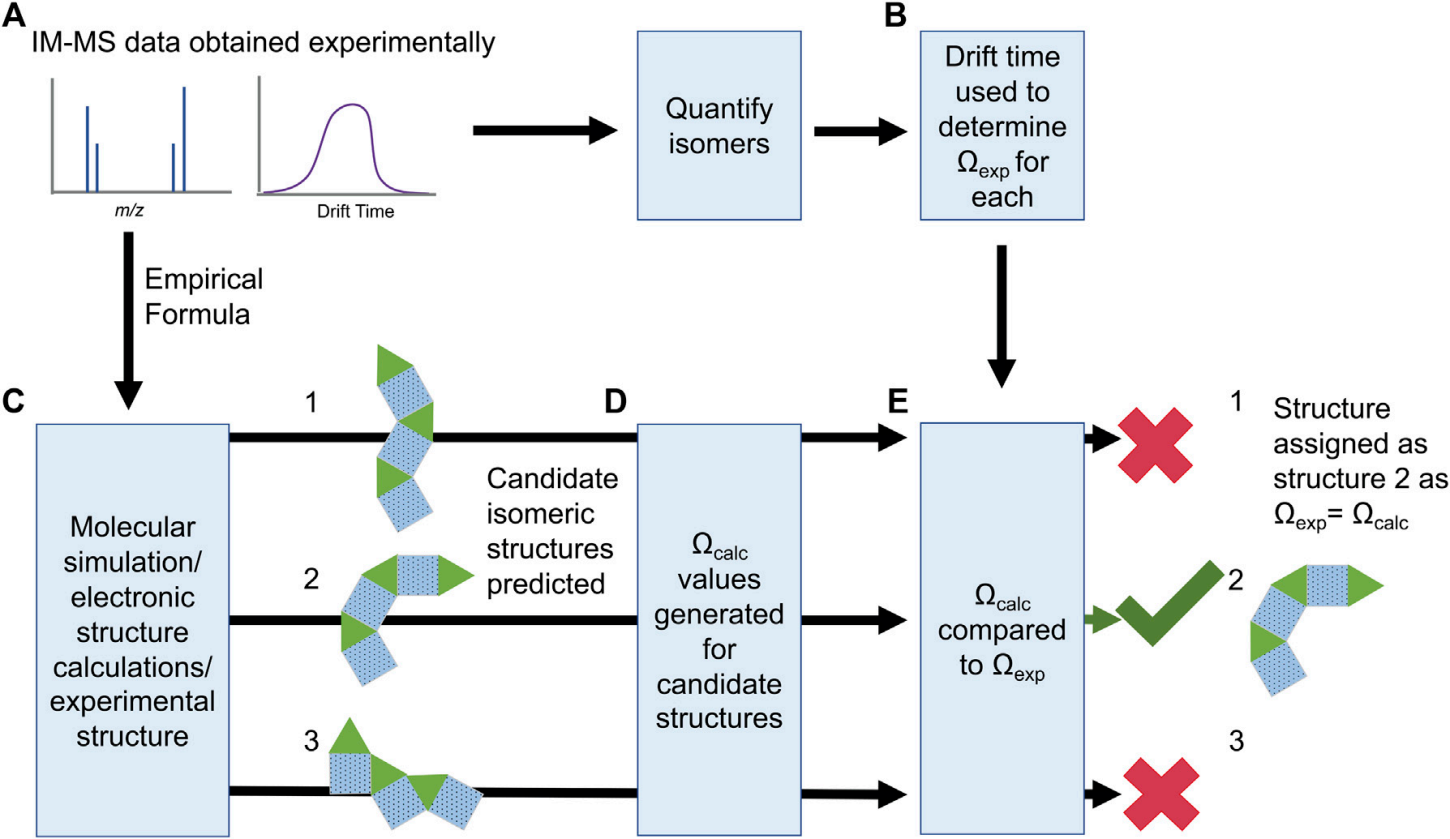
IMS-MS data can provide **(A)** experimental drift times and masses. These can be converted to **(B)** Ω_exp_ values and **(C)** empirical formula for structure prediction. The predicted structures can then have **(D)** Ω_calc_ values predicted and compared to the Ω_exp_ values to give **(E)** confident structural assignment of the observed ions.

Candidate structures for IMS-MS are typically predicted using computational methodologies, such as molecular simulation or electronic structure calculations; recent in depth reviews on combining these techniques with IMS-MS are available (Lapthorn et al., 2013; D'Atri et al., 2015; Boschmans et al., 2016; Eschweiler et al., 2017; Indelicato et al., 2017; Konermann et al., 2018; Marklund and Benesch, 2019; Prell, 2019; Rolland and Prell, 2019). These predicted structures are then used as inputs for a collisional cross section calculation. Importantly, a calculated collision cross section is only as good as the underlying structural “guess” the cross section is computed from. Confident structural assignment is achieved when predicted collisional cross section values for candidate structures (Ω_calc_) are compared and matched with experimental Ω_exp_ values (Figure 5), usually in units of Å^2^. While there is always error involved in both calculation and experiment, in some cases of rigid structures with a finite number of distinctive isomers, unambiguous structural assignment can be achieved.

Of the classical approaches, molecular dynamics (MD) simulates the overall force an atom in a system experiences through the sum of approximated bonded and non-bonded interactions(Durrant and McCammon, 2011). In contrast, Monte Carlo (MC) molecular modelling generates new system states based on a Boltzmann distribution. A benefit of MD and MC is that both can be scaled to “coarse grain” models rather than atomistic when the analyte systems prove too large for efficient computation (Vangaveti et al., 2017). As the approximations are relatively computationally inexpensive, efficient simulations to probe the dynamics of even very large assembled systems and supramolecules are possible. Subsequently the most likely structural candidates predicted by MD will be used as input structures to calculate Ω_calc._ However, its not guaranteed that MD will find all available energy minima, potentially leading to overlooked structures. Many MD force fields are optimised for the aqueous phase and so care should be taken to compensate for this effect if predicting gas-phase structures (D'Atri et al., 2015). Similarly, many force fields are not parametrised for use with transition metals (Bursch et al., 2021). However, these methods offer great insight into dynamics of a system not accessible via other methods.

Electronic structure calculations include well known computational methods such as density functional theory (DFT) and ab initio calculations. These methods can optimise conformational structures and accurately predict relative energies. This allows for determination of lowest energy isomers and conformers. One challenge is sampling the full conformational landscape to ensure that the structures optimised represent the full breadth of structures available, which can be difficult in certain systems with transition metals (Bursch et al., 2021). Careful manual or automated conformer generation, termed conformational search, is required. Electronic structure calculations represent a bottom-up approach to structure, which often leads to very accurate structures for small molecules, yet can be too computationally demanding for larger systems (Boschmans et al., 2016; Gunzer, 2016).

A third viable method for obtaining structures is to use information obtained by crystallography or NMR (Jurneczko and Barran, 2011). Whilst providing a quick and accessible option, the use of solid or solution phase structures as an approximation of gas phase structure might lead to significant error in Ω_calc_. A better option is to use these structures as starting input structures for further optimisation using one of the methods outlined above (D'Atri et al., 2015).

Both molecular simulation and electronic structure methods have been used to obtain structures for coordination self-assemblies (Brocker et al., 2010). Sometimes both are used in tandem, using molecular dynamics to obtain a “rough” structures and follow up with DFT to obtain accurate energies and optimised structures (Warzok et al., 2018). No matter the method used to obtain the optimised structure(s) the collision cross section area must then be calculated (Figure 5D). There are several software packages that implement a range of calculation methods, including but not limited to the trajectory method, projection approximation and exact hard sphere scattering (Mesleh et al., 1996; Shvartsburg and Jarrold, 1996; Marklund et al., 2015; Ewing et al., 2017). Different methods and software implementations (e.g., MOBCAL, IMPACT and Collidiscope) confer different benefits and in-depth comparisons can be found elsewhere (Marklund et al., 2015; Prell, 2019) Many methods systematically under or overestimate Ω_calc_ and so sometimes multiple methods are used to strengthen an assignment (Chan et al., 2011; Ebbert et al., 2019). An Ω_calc_ value that matches Ω_exp_ within error after this process allows confident structural assignment of a theoretical structure to an observed ion (Figure 5E). Where several possible matches are possible, relative energetics and mechanistic insight from the calculation can often eliminate unreasonable Ω assignments.

### Mobility Resolution

The ion mobility resolution is an important facet of each technique to consider, defined as the smallest change in mobility that can be detected by the technique. The theory of ion mobility resolution borrows in some respects from the theory of chromatography; the historical name of “plasma chromatography” belying the way it was originally considered (Revercomb and Mason, 1975). For DTIMS exclusively, there are helpful parallels to plate-height models that can be drawn, however it is inaccurate to attempt to characterise the separation of ions in ion mobility as a chromatographic process (Grabarics et al., 2020). Resolution is usually assessed through either a single peak or two peak method. Recent efforts have been made to harmonise these for comparison across techniques (Dodds et al., 2017). In general, the standard low-field ion mobility techniques were found to readily separate analytes with Ω differences in the order of 1% and above, but modified instrumentation such as Structures for Lossless Ion Manipulations (SLIM), TWIMS or High Pressure DTIMS is required to consistently resolve below 1% (Dodds et al., 2017). Analysts should keep the resolving power of their instrument in mind when considering structurally similar analytes, especially the sometimes subtle structural differences in assembled structures and intermediates, such as isosteric species (isosteric species being those that have the same or similar shape and size, and the same number of valence electrons arranged in a similar manner). Throughout this review there are numerous examples of the low field ion mobility techniques described resolving very similar structures.

### Ionisation Techniques

The ionisation techniques used to analyse CDSA systems are typically “soft” techniques that can preserve the intermolecular bonds that define supramolecular chemistry (Henderson and McIndoe, 2005; Schalley and Springer, 2009). As self-assembly is typically carried out in solution, electrospray ionisation (ESI) or nano-ESI (where the flow rate, orifice size and droplet size are reduced) are commonly used. Though consideration must be given to how non-covalent bonds change in the gas phase (Schalley and Springer, 2009; Konermann et al., 2013), decades of work indicate ESI yields ions representative or at least indicative of species present in solution. ESI can generate multiply charged ions, which benefit analysing larger assemblies (and biological assemblies such as proteins) as the increased charge lowers the *m/z* value inside the mass range accessible. Highly charged ions’ masses can be deconvoluted to give accurate mass values (Bern et al., 2018).

One of the challenges IMS-MS faces when applied to CDSA is discerning structures representative of solution behaviour from gas phase structures. One of the downsides of ESI might be the production and destruction of aggregates that may not necessarily exist in solution. Oxidation and reduction can also be induced, changing the charge state observed from that present in solution. Investigating the tuning of ionisation conditions to produce adduct free complexes revealed that counter anions have a stabilising effect on CDSA complexes, allowing observation of fragile products, which may impact the choice of counter anion (Mallis et al., 2019). Without counter ions, CDSA products are likely to experience greater electrostatic repulsion interactions, leading to lower stability. This same work also demonstrated that counterions can cause changes in Ω (i.e structure).Thus the ionisation process and gas phase behaviour gives hitherto unavailable insight into potential stabilisation that might be occurring in solution. In a similar vein, the choice of solvent for electrospray can influence the structure. Typically, polar solvents like methanol and water, or acetonitrile are used ESI, which limits which assemblies can be analysed to polar assemblies. Different solvents being employed with ESI can show clear switching of structures observed, one example showed changing the solvent for both reaction and electrospray between dichloromethane and chloroform switched the structure reversibly from hexamer to a pentamer (Warzok et al., 2018); again showing how electrospray gives insight into solution phase behaviours not generally accessible by other methods. A further limitation is the need to use dilute solutions (<1 mM), which again, may not be typical of the equilibrating solution under analysis. However, one of the strengths of ESI is the ability to observe kinetically trapped intermediates, by “pausing” the solution phase equilibria when it is transferred to the gas phase.

Another soft ionisation technique often used is Matrix Assisted Laser Desorption/Ionisation, MALDI (Zenobi and Knochenmuss, 1998; Frischmann, 2010; Yin et al., 2018). MALDI is especially powerful for allowing the analysis of analytes that are large and/or poorly soluble and thus analytically inaccessible via ESI. One of the disadvantages of MALDI is the presence of dopants in the data collected, which may complex with these types of analytes (Wyatt et al., 2008). Due to the sample plate method of loading, it is also typically a static, batch based workflow, less amenable to reaction monitoring. Atmospheric Pressure Chemical Ionisation, (APCI), whilst a soft ionisation technique, has not been applied to analyse CDSA supramolecules directly, but has been applied to analyse the precursor ligands (Frischmann, 2010; Jansze, 2019).

### Mass Analysers

A range of mass analysers are used in tandem with ion mobility spectrometers. The main consideration when hyphenating the two techniques is the relative cycle time of each instrument. If the cycle time for MS detection is not sufficiently fast, then ions separated by ion mobility will not be detected separately. Thus, the cycle time of the mass spectrometer should be shorter than the arrival time difference between two separated ions. Time of flight (ToF) analysers are a natural fit for their very fast cycle times (which are only improving with advances in electronics), and many of the commercially available IMS-MS systems use ToF analysers. For mass analysis, quadrupole analysers are not often used in conjunction with ion mobility, due the relatively slow acquisition rate and low resolution (especially in comparison to ToF). However they are often used as mass filters and collision cells (Adamov et al., 2007). Despite the relatively slow cycle time (again, in comparison to ToF) of trap-based spectrometers, many have recently been combined with ion mobility successfully, including 3D ion traps (Donohoe et al., 2014), Orbitraps (Ibrahim et al., 2016), and Penning traps (Fourier-transform ion cyclotron resonance, FTICR) (Ridgeway et al., 2018). There are now commercial IMS-MS options available for these mass analysers. This has enabled the collection of very high mass-resolution (high resolution mass spectrometry, HRMS) data without sacrificing ion mobility separation. However, due to the IMS separation of charge states and other isobars, when analysing CDSA systems, the need for very high mass resolution can often be circumvented.

### Tandem Instruments

One final aspect to consider when discussing IMS-MS is the specific configuration and dimensionality of the instrumentation. Tandem gas phase experiments can be applied in any sequence before mass analysis. For example, quadrupole mass filters before IMS separation allow for mass selection and fragmentation, and the mobility of isolated species and fragments to be analysed. Penning trap (FTICR) and tandem MS instruments combining a quadrupole or ion trap with a high-resolution mass analyser are commercially available, enabling MS^2^ or MS^n^ experiments followed by accurate mass measurement of the fragments produced. Similarly, cyclic ion mobility devices and tandem ion mobility instruments provide possibilities for IM^n^ options, which can be employed to study changes in shape over time (Giles et al., 2019). This variety of instrumentation allows the analysts to customise their approach to analysing coordination driven self-assembly, and the dynamics of the assemblies produced.

## Applications of Ion Mobility Mass Spectrometry to Study Coordination Driven Self-Assembly

### Separation Data

The simplest and most intuitive application of ion mobility mass spectrometry to CDSA is in the separation of distinct self-assembled products. This allows “clean” mass spectra of individual ions to be recorded in the absence of interfering ions. When applied like this the imperfect comparison to chromatography can be made, as ions are “eluted,” and the total ion count analysed in turn. But the entire “elution” occurs over milliseconds. This is powerful, as actual chromatographic methods may cause transformative stability issues with supramolecules, akin to the unfolding of proteins (McNay and Fernandez, 2001), and have significantly slower cycle times.

**FIGURE 6:**
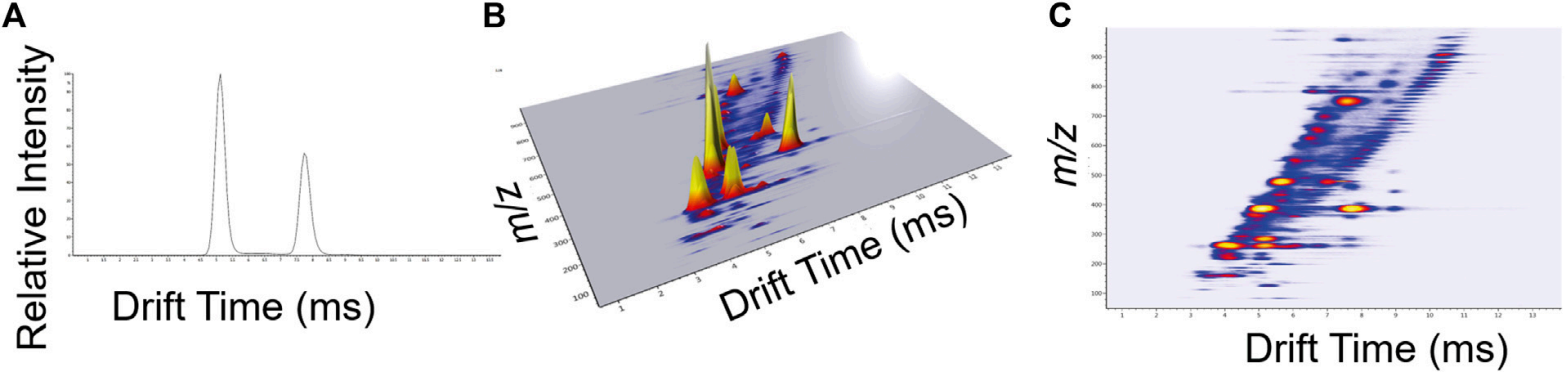
Representative IMS-MS data visualisation of metal-ligand mixture (iron chloride and deuterated acetylacetonate): **(A)** a typical ion mobiligram drift trace (drift time vs. intensity) for *m/z* 395; **(B)** a 3D intensity map plot where the height of a peak shows the relative intensity of an ion with a particular arrival time (note in print certain peaks are obfuscated); **(C)** an intensity heatmap where signal intensity is mapped to colour (presenting the same data as **(B)**). Spectrum is ESI-TWIMS-QTOF.

Ion mobility mass spectrometry data are presented as an arrival time distribution, also known as mobilograms, plotting drift time vs. ion intensity for a particular m/z (Figure 6A). These individual mobilograms may be built into a 3D intensity map plot covering over the entire m/z range analysed (Figure 6B). The data can also be visualized as a heatmap of drift time vs. *m/z*, where peaks represent the ions observed at a particular time in the experiment, and colour represents intensity (Figure 6C). In the case of TIMS data, inverse reduced mobility values are reported instead of drift time, as ions are not resolved by drift time (Figure 4).

### Separation of Charge States in Coordination Driven Self-Assembly

**FIGURE 7:**
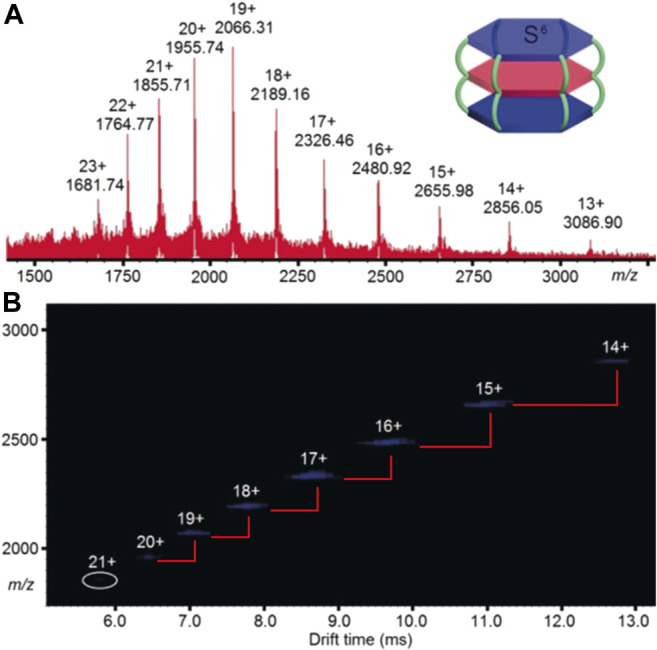
Comparison of **(A)** ESI-HRMS and **(B)** ESI-TWIMS-MS data showing the distinct charge state step change, highlighted with red lines showing change in time and *m/z*. Note the step change is not a constant value because the effect of a single charge difference increases as charge decreases (Liu et al., 2018).

Ion mobility spectrometry efficiently separates ions by charge state. Thus ions of the same molecular mass but different overall charge upon ionisation will possess different mobilities. This is highly beneficial for large assemblies, where it is often impossible to obtain high resolution mass spectra with clear isotope patterns. A key feature observed in heatmap plots is the “step change” between ions that represent the same supramolecule with different charge states. This occurs because the change in charge effects *m/z* and k (*k* ∝ Ω/*z*, Eq. 2) by approximately the same amount, leading to the distinct pattern. This is illustrated by the arrival time distribution of a triple decker, spoked wheel CDSA structure (Figure 7B). The synthesised structure’s mass of 42,014.3 Da is very large, and able to be transferred to the gas phase by ESI. However, despite ESI beneficially conferring multiple charge states, the combination of the assemblies large size and high charge state does not allow for the isotopes to be resolved by HRMS. Thus, ion mobility is needed for structural confirmation. The *m/z* signals are sharp, and the drift time signals discrete (i.e., not bleeding into one another). This distinctive “step change” pattern confirms the proposed structural homogeneity in the final CDSA product, with no unexpected fragments or misaligned structures (Figure 7) (Liu et al., 2018). In this case IMS-MS is confirmatory of the absence of other structures, i.e., the spectra are “clean.”

### Separation of Isobars and Confounding Isomers in Coordination Driven Self-Assembly

Ion mobility can also filter confounding isomers and isobars. Isobars are defined by IUPAC to be ions that have the same nominal mass but different exact mass (Kermit et al., 2013). However this definition can be expanded to include oligomeric charge state series ions with the same exact *m/z* corresponding to structures differing by *n*-repeating subunits of charge *n*. These share the same exact *m/z*, but possess different molecular masses. Charge state series can often be identified in MS by isotope patterns, but these rely on the subunits possessing isotopes, along with both adequate sensitivity and resolution of the MS, especially for large assemblies. Thus when a homologous series of assemblies are synthesised within the same mixture, these are often isobaric (Figure 3).

**FIGURE 8:**
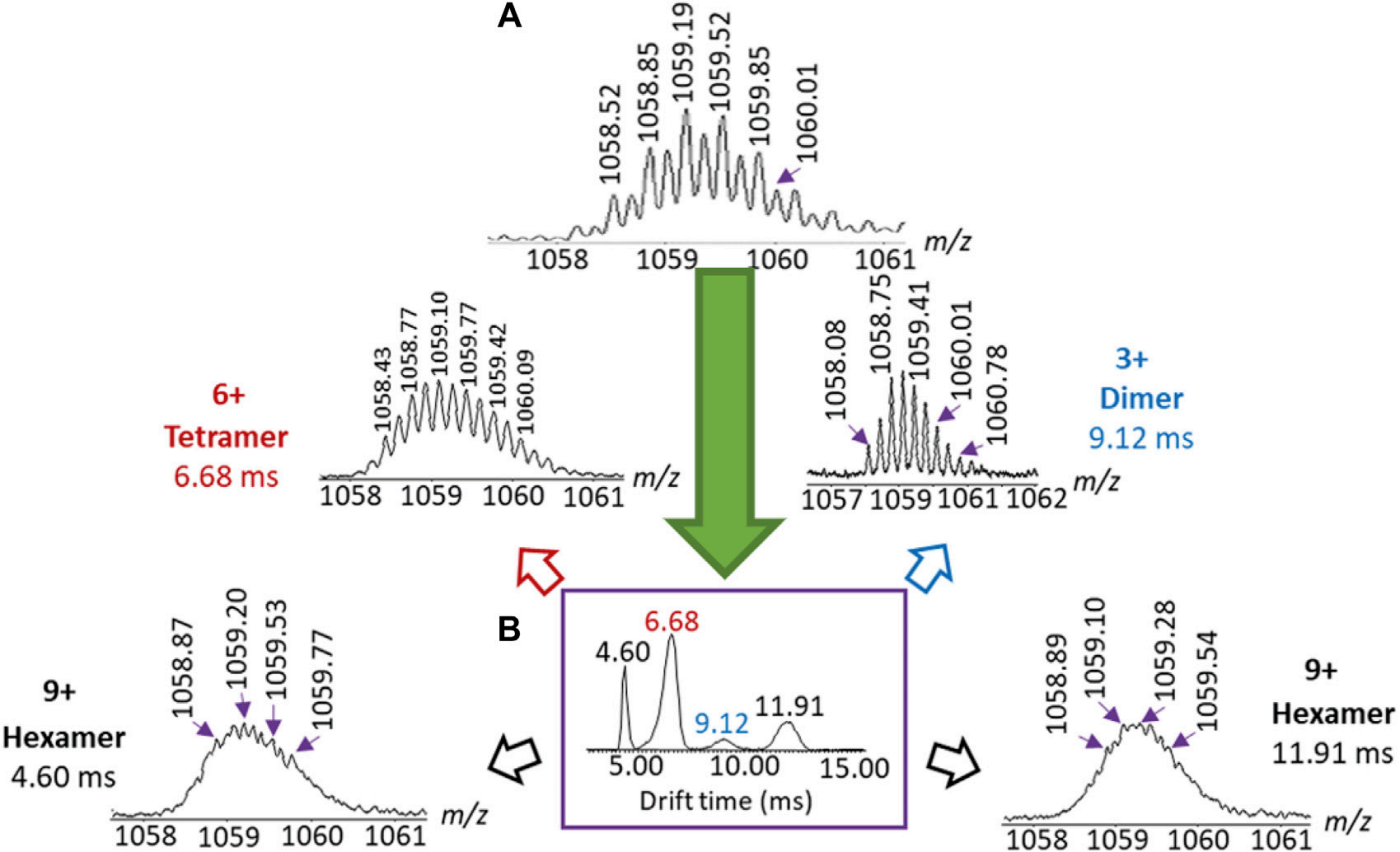
IMS-MS separates isomers and isobars and then identifies them by both *m/z* and drift time. The data shows **(A)** multiple MS signals around *m/z* 1,058 which can be separated by IM to give **(B)** four peaks are observed in the ion mobiligrams, two of which can be assigned to the 9^+^ linear hexamer and the cyclic hexamer as well as two other isobaric 6^+^ and 3^+^ cyclic isobars Note the isobaric species are all 3*n* ^+^, as they correspond to the 3*n* homologous series (Endres et al., 2020).

A common example of isomers is the presence of both of linear and polyhedral isomeric assemblies that incorporate the same repeat units, thus giving them *m/z* values that overlap despite having wildly different structures, a problem also seen in traditional polymerisation (Hoskins et al., 2011; Endres et al., 2020). CDSA systems often use ligands designed to limit linear isomer formation by taking advantage of rigid π-connectors and directional metal-ligand bonds to create angles between binding centres. Despite this, these linear complexes are still often observed for angled ligands (Figure 3). For example CDSA complexes have been synthesised incorporating either 120° “bent” or 180° “linear” isomeric ligands (where the angle denotes the angular difference between the two metal binding sites) (Li et al., 2011a). The ion mobility separation of the linear complexes were compared to those of the bent complexes and revealed the presence of analogous linear assemblies formed by the bent ligand. TWIMS was able to partially resolve these assemblies and identify them. By comparison, almost a decade later, another set of libraries were prepared with a bent ligand alone and TWIMS successfully identified multiple isobaric and isomeric components, including linear isomers, without the preparation of a secondary linear library (Figure 8) (Endres et al., 2020). Similarly, cyclic isobaric assemblies formed by dissymmetric ligands were recently reported to be separated by TWIMS-MS (Shi et al., 2021).

**FIGURE 9:**
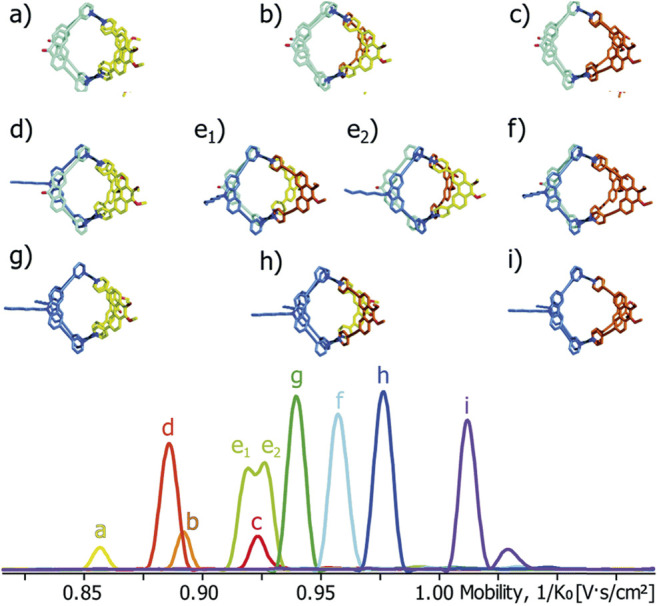
Heteroleptic Palladium cages **(A–F)** separated by TIMS. The cages observed represent the observed self-assembly products of four bis-pyridine ligands with similar structures. Note that these cages could all be identified by mass differences, except *cis*/*trans* isomers e_1_ and e_2_, which were only separated and identified by TIMS.

Another impressive example of the separative power of ion mobility was shown in the separation of a family of 10 heteroleptic CDSA cages (Figure 9) (Ebbert et al., 2019). Here it was demonstrated that TIMS-MS could separate and resolve the statistical mix of cages created by four ligands efficiently. The ligands were non-isomeric, but structurally similar, and the subsequent cages had Ω values that differed by ∼4% (approx. 20 Å^2^). Not all cages were baseline resolved by TIMS, however, could be distinguished by MS. Perhaps the most exciting result was the partial separation and identification of *cis* and *trans* isomers, whose Ω values differed by 0.8%. These results also highlight that IMS-MS can be used beyond separation to gain structural understanding.

### Structural Identification

Using IMS-MS to simply separate isomers and isobars is the tip of the iceberg. As previously discussed, the relationship between Ω and *K* can be linked through Eq. 2. As such, quantitative values for both the mobility coefficient (*K*) and the collision cross section (labelled both as Ω and CCS) can be obtained. Experimentally obtained Ω is complicated and difficult to intuitively interpret, though structural trends revealed can be meaningful (e.g., more compact vs. more open molecules). More powerful is comparing predicted Ω of modelled candidate structures Ω_calc_ with experimentally derived Ω_exp_ to assign structures (Figure 5).

One early demonstration of this approach being applied to the field of CDSA was reported from the Bowers group (Brocker et al., 2010). DTIMS was used to record the arrival time of several platinum-pyridyl species, including both final multimeric assemblies and isobaric intermediates. The Ω_exp_ values were then calculated using Eq. 2. These experimentally obtained values were then compared to values calculated using two separate modelling methods. The input structures for these calculations were based on crystal structure coordinates. The strong agreement between known structures obtained from crystallography and ion mobility, subsequently allowed the assignment of other structures, which had not yet been confirmed by crystallography.

Another example of careful comparison between modelled and experimental cross sections is demonstrated by the structural determination of metalloporphyrin oligomeric assemblies (Schwarz et al., 2013; Schwarz et al., 2014). Interestingly, as hydrogen counterions were exchanged for sodium counterions, topological changes of the dimer were observed using TWIMS (Schneider et al., 2018). The counterintuitive contraction of the dimer assembly occurred as the number of sodium ions increased. The increase in interaction strength led to an overall compaction, which compensated for the size increase due to additional counterions. The observed changes in Ω_exp_ were compared to Ω_calc_ values obtained from trajectory method applied to carefully optimised DFT structures. This built on similar earlier work observing the change in oligomeric metalloporphyrin geometry as the coordinating metal was changed (Brendle et al., 2016). It was found that for M^II^ containing oligomers, the charge state of the oligomer, and the corresponding number of sodium counterions, dictated if the structure formed would be “open” or “closed.” Interestingly, M^III^ containing oligomers did not exhibit the same pattern, as the sulfonic groups of the porphyrins could readily bind to the central metals. It could be interesting to reduce or oxidise the metal centre incorporated in the metalloporphyrin and to observe the potential structural transformation by IMS-MS (Marshall et al., 2020).

**FIGURE 10:**
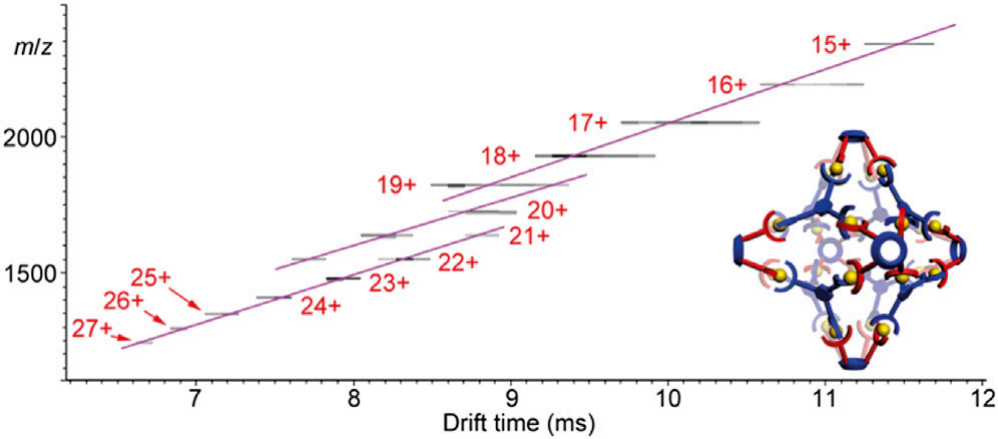
Observation of two distinct cubic star structures seen in ion mobility dependent on assembly charge. These two structures are represented by the two different families of peaks (pink lines) observed with a smaller transitionary family observed in between (He et al., 2020).

An exciting example of structural assignment using Ω was reported in the synthesis of multiple supramolecular cages based on Calixresorcinarene ligands (He et al., 2020). Three cages were formed with ligands that varied only in the length of the π-spacer groups. These cages were interrogated by calibrated TWIMS-MS and obtained Ω values were averaged and compared to values calculated using the trajectory method with MOBCAL. Whilst the smallest two cages showed relatively standard “step change” data, the largest cage gave two distinct patterns, due to the increased flexibility of the spacer. This allowed the cage to compensate for electrostatic interactions with a shape change at higher charges. This is a direct observation by IMS-MS of a charge effect on structure. When analysing a similarly synthesised cubic star structure, a similar effect was observed (Figure 10). Further analysis of the high charge and low charge structures via Ω_calc_ could give insight into the impact of the counterion stabilisation of charge on the supramolecular structure. Shape changes can be exploited to give supramolecular assemblies functionality; thus, a systematic understanding of the charge induced shape change could prove useful for future synthetic efforts.

Mass spectra provide a wealth of structural information for investigating products and intermediates of CDSA, through mass, isotopes, charge states and fragmentation patterns (Hopfgartner et al., 1994; Brodbelt and Dearden, 1996; Schalley, 2000; Schalley, 2001; Baytekin et al., 2006; Weimann et al., 2012; Cera and Schalley, 2014; Warzok et al., 2019). However, Mallis et al., have observed that the high rotational symmetry of many CDSA products lead to precursor and the product ions of collisional activation being obscured in the mass spectrum in many cases (Mallis et al., 2019). When combined with IMS, MS^2^ and MS^n^ experiments can give even more specific structural insights. For example, product ions formed via collision induced dissociation (CID) can be further identified via their Ω (Li et al., 2011a), and isomeric and different charge state fragments can be separated. Thus, the diversity of potential structures formed by CDSA can be easily assessed using MS and IMS-MS, combining the strengths of both techniques (Yu et al., 2018). Despite this potential, MS^2^ data in combination with IMS are less reported in the current literature than might be expected, a surprising observation. IMS-MS can separate these isobaric ions, but this ability is dependent on the availability of an ion mobility region after the collision region of the instrument. MS^n^ may see a resurgence in reporting (even in the absence of IMS) if the current synthetic focus on heteroleptic cages is maintained, as these will exhibit a decreased rotational symmetry due to presence of different ligands.

Whilst there is huge opportunity to use IMS-MS to discern structural features, this aspect of the technique is currently under employed. This could be due to a perception that ion mobility is a purely separative technique akin to chromatography. It could also be because TWIMS requires calibration to obtain Ω values and this can be challenging when observing novel systems where calibrants of a similar size and nature should be used (Wang et al., 2014b; Mallis et al., 2019). Pleasingly, more diverse calibrants, such as inorganic calibrants, are being added to collections of known IMS calibrants (Surman et al., 2016; Hupin et al., 2018). Additionally, Ω values are most meaningful when compared to theoretical structures to look beyond “trends,” which can require calculations that may be beyond the time or scope of a given study.

The complementarity of IMS-MS when added as a characterisation technique cannot be overstated, with numerous recent examples of structural agreement between IMS-MS, x-ray crystallography and NMR/DOSY NMR, TEM etc. (Chan et al., 2009; Brocker et al., 2010; Chan et al., 2011; Lu  et al., 2014; Wang et al., 2014a; Wang et al., 2014c; Bonakdarzadeh et al., 2015; Liang et al., 2015; Xie et al., 2015; Li et al., 2016; Xie et al., 2016; Wang et al., 2018a; Wang et al., 2018b; Zhang et al., 2018; Jiang et al., 2019; Sarkar et al., 2020) Indeed, recent examples have shown, adding IMS-MS to the characterisation workflow can help avoid missing products due to, for example, a lack of crystallization (Christie et al., 2016).

Recently IMS-MS has not only characterized occluded CDSA products, such as constitutional isomers and configurational isomers (Ujma et al., 2012; Schultz et al., 2013; Liang et al., 2015), but also the “erroneous” intermediates formed in multicomponent systems (Wang et al., 2020c), or impressively, the lack thereof in a multicomponent system, due to successful pre-organization (Wang et al., 2020a), and even the formation of pre-organized “sequenced” CDSA systems (Song et al., 2018). As such, the structural analysis of CDSA systems using IMS-MS ultimately leads us beyond characterisation of the product and side products, and towards an understanding of CDSA mechanisms.

### Structural Dynamics and Reaction Monitoring

By measuring an ion mobility spectrum as conditions such as reactant concentration, reaction time and temperature are varied, structural changes and intermediates can be revealed. This is important as the dynamic nature of self-assembly means some qualities of the system can only be observed when conditions are perturbed or monitored with time (Lanucara et al., 2014).

**FIGURE 11:**
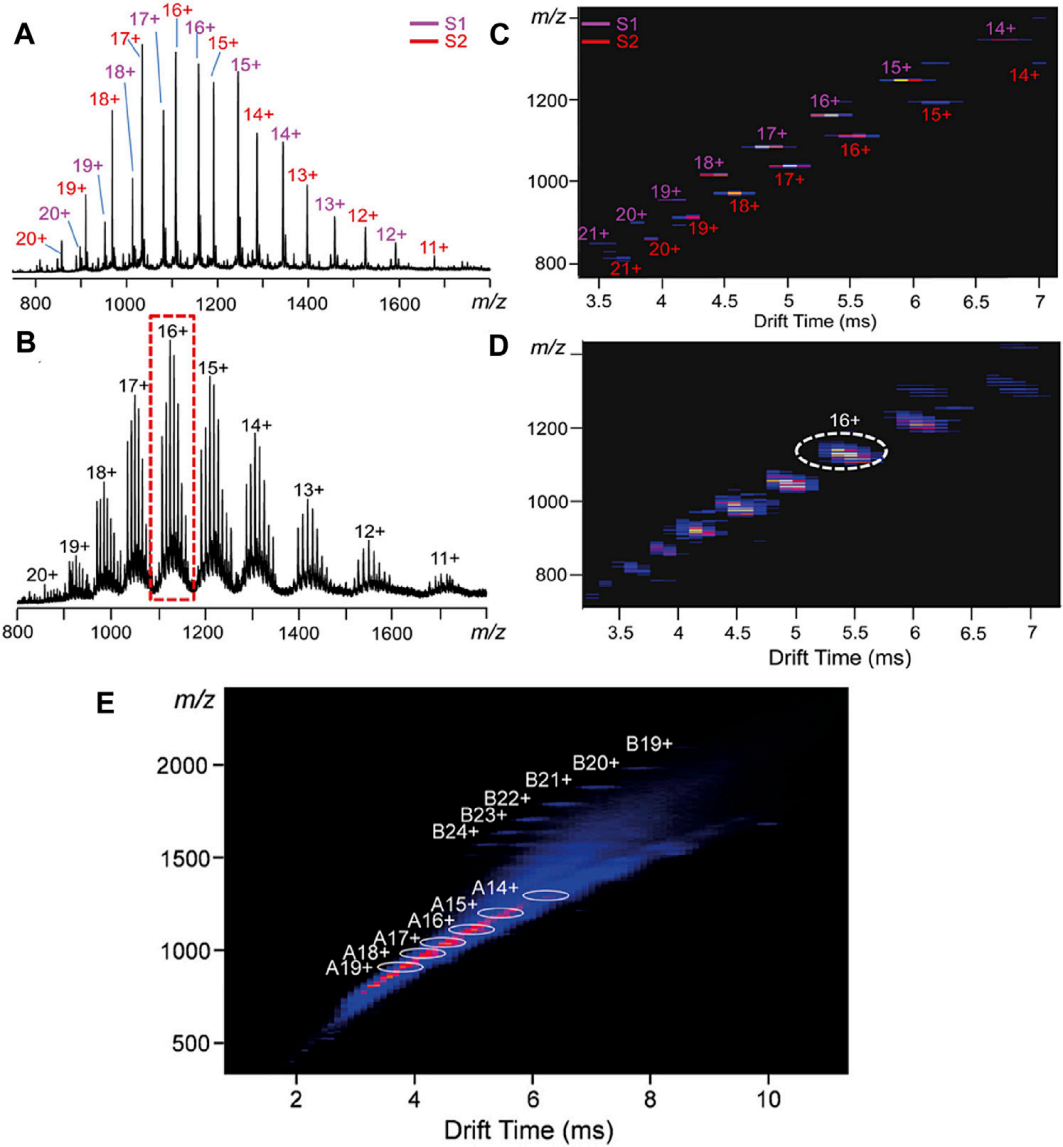
IMS-MS allows the observation of intermediates in dynamic processes. The mixing of two snowflake assemblies over 5 days shows a clear change from **(A)** two clear series of distinct peaks at time = 0 to **(B)** 7 peaks on day 5 representing the population of a statistically mixed set of ligands. Additionally, this change is visible in the change in the ion mobility heatmap where **(C)** two clear chains of ions a time = 0 become **(D)** mixed to create a larger peak positioned between the two chains (yellow circle) (Zhang et al., 2017). In other work, two clear sets of IMS-MS signals denote a lower *m/z* series of intermediates at different charge states (A-series) and a higher *m/z* series of final assemblies at different charge states (B-series) (Wang et al., 2019).

Time is a critical variable in coordination driven self-assembly. As most reported CDSA equilibria are thermodynamically driven, the first products observed may be thermodynamic intermediates. Structural time dependency can be seen in the analysis of two terpyridine based “snowflake” CDSA systems (Zhang et al., 2017). Two preassembled supramolecular snowflakes were mixed and both the mass spectra and ion mobility drift time distribution show initial preservation of two unique assemblies at *t* = 0 (Figure 11A). However, after 5 days, the solution shows a statistical mixing of the two systems, observed through additional ion peaks of intermediate mass in the mass spectrum, and the “blending” of previously distinct ion mobility signals (Figure 11B). This shows that the starting structures are not the thermodynamic endpoints of this mixed system, i.e., they are not self-sorting. The dynamic exchange between the two snowflake supramolecules and the approximate timescale over which it took place were successfully observed. Similar results were reported in the study of solution phase dynamic ligand exchange of the two ligands’ respective supramolecules. By measuring the change in concentration of the two complexes by IMS-MS over 22 h, the linear change in complex concentration was recorded and second order rate constants were determined for the process (Wang et al., 2020b).

The intermediates during the formation of metallo-supramolecular hexagonal prisms have also been clearly revealed by ESI-TWIMS-MS measurements (Wang et al., 2019). The intermediates were formed in a milder version of the self-assembly reaction, simulating the reaction mixture, before the final product is formed. The ESI-TWIMS-MS data showed two clear sets of complexes formed, marked A and B in Figure 11E. The lower *m/z* complex was assigned to the complex representing a single hexagonal “face” of the prism. Additional intermediates were detected by MS, corresponding to additional coordinating cobalt ions. These intermediates were used to propose a mechanism, but assigned molecular formulae give no indication as to the intermediates’ structure. The intermediates could not be unambiguously confirmed as hexagonal face as posited by the authors. Whilst the proposed mechanism is both sensible and plausible, further supporting evidence could allow it to be confirmed. Again, the mass of the assembly investigated in this work was >40,000 Da, highlighting the impressive power of ESI to volatilise high mass structures and create ions with an *m/z* within the workable mass range of mass analysers.

Time is not the only factor that can affect the self-assembly products observed, concentration is also critical. For example, serial dilutions of a CDSA mixture in solution, zinc with tetrakis-terpyridine ligands, gave rise to the formation of three different polyhedral assemblies, which were monitored by TWIMS-MS (Endres et al., 2019). A single *m/z* that contained isobars representing each of the three structures was monitored as concentration was changed and the results were used to find equilibria constants for conversion between the assemblies. In this case a clear dependence between ligand concentration and the position of the equilibria, and hence the structure formed, was observed. A single *m/z* was chosen due to the presence of three resolvable isobars at measurable abundances. A potential weakness of this approach is that it does not account for the potential charge state preferences of each structure. Regardless, this work presents a powerful methodology for studying the effect of ligand concentration on structures that are produced by CDSA, despite the obfuscation of supramolecular concentration caused by isobaric overlap.

Another example of ion mobility being employed to determine the effect of changing concentrations on CDSA structures can be seen in the reversible conversion of a cage to a ring (Jurcek et al., 2015). This was driven by changing the concentration of chloride ions present in solution. As chloride is added to the solution, it preferentially coordinates to the palladium metals of the cage and the bis-pyridine ligands are substituted through a series of intermediates, eventually leaving a cyclic structure. If chloride metals are removed from solution the reverse was observed, as is expected for self-assembly, and confirmed the effect of the chloride ions. The change in structure could be tracked by the changes in Ω and *m/z* observed by DTIMS-MS and was confirmed with ^1^H DOSY NMR. An isomer of the cycle and dimeric structures were prepared; however, their IMS-MS data was not reported. A comparison of the two isomeric cycles could potentially have helped confirm the results of the prior work. This excellent work demonstrates the power of IMS-MS to monitor reactions, as the shape change can be directly linked to the progress of the reaction.

Changes in solvent can cause dynamic changes in assembly structure. Warzok et al. synthesised capsules through coordinative halogen bonds (Warzok et al., 2018). As the solvent for both reaction and electrospray was varied between chloroform and dichloromethane, the structures of the capsules changed from hexamers to pentamers. Both these species were characterised by IMS-MS, with calculated and experimental Ω values matching well. This was further probed by a range of mixed solvent solutions, revealing the structural change in proportion with solvent proportions. A collision induced dissociation (CID) experiment was employed to demonstrate this was a solution phenomenon and not a result of gas phase rearrangements.

Another important parameter when utilising coordination driven self-assembly is the reaction temperature. This can be another tool to control the selectivity and reactivity; the outcome of a reaction. To get an idea of the relative energetics of a reaction, the relative barriers can be probed in the gas phase via low energy CID. Energy resolved CID is the application of different collisional energies to the same ion. By varying the collision cell potential over a range of voltages, the energy imparted upon collision with a buffer gas is varied, in turn varying the unimolecular isomerization and dissociation pathways (Colorado and Brodbelt, 1996). This allows for relative energetics of these pathways to be compared (while specialised apparatus can even yield absolute threshold energies (Armentrout, 2003)). Gradient MS (gMS^2^) utilises energy resolved CID in tandem with IMS (Wesdemiotis, 2017). The ion mobility separation of the subsequent fragmentation products highlights structural changes precipitated by CID. Sometimes gMS^2^ is defined independent of ion mobility but we recommend that future use of the definition includes ion mobility performed immediately before or after fragmentation (Li et al., 2011b; Zhang et al., 2017). gMS^2^ provides stability information for observed species, with species fragmenting as the voltage is increased and their individual threshold dissociation energies are met (Colorado and Brodbelt, 1996). The data are typically presented as multiple mobiligrams against a collision voltage axis, showing clear changes in fragment abundance with voltage. One recent example of gMS^2^ allowed for the characterisation of a supramolecular isomeric fragments where one fragment is more unfolded than the other (Guo et al., 2015). gMS^2^ represents one of the most exciting implementations of IMS-MS as a structural technique, as it allows one to probe structural dynamics. Earlier work was reported varying the ion injection energy, causing increased in-source fragmentation, the product ions of which were then detected and tracked by DTIMS and correlated with energy to reveal the thermal stabilities of assemblies and their components (Brocker et al., 2010).

Additionally, varying the temperature of the drift tube gas, a technique pioneered by Bowers, Jarrold and others, could provide further opportunity for developing the structure of gas phase CDSA ions (Jarrold et al., 1989; Kemper and Bowers, 1990). The benefits of lowering the temperature include reducing thermal diffusion, narrowing the arrival time of a given ion, resulting in thinner, more resolvable peaks (May and Russell, 2011). An example of this is seen in Brocker *et al.*, where isobaric fragment ions with the same arrival times at 300 K could be differentiated at 77 K (Brocker et al., 2010). Additionally, by lowering the temperature of the drift gas analyte ions can be cooled and kinetics can be slowed, “freezing” molecules in conformations, stopping interconversions, and allowing the resolution of two different species by IMS-MS. By increasing the temperature, ions gain increased thermal energy, encouraging conformational interconversion which may allow them to access conformations that they could access in solution but lose access to in the gas phase (Wyttenbach et al., 2014). These experiments provide a fresh opportunity to interrogate the molecular dynamics of CDSA supramolecules.

### Increased Levels of Structural Complexity

Whilst there is no formal delineation between levels of structural complexity as in molecular biology, self-assembled structures can move beyond the ubiquitous rings and cages to a higher level of structural complexity, some termed “molecular machines” (Gómez-López et al., 1996). Fundamental to the ability to synthesise many “molecular machines” are the catenane, rotaxane and other mechanically bonded motifs (Balzani et al., 2000). Mechanical or topological bonding entangles two or more discrete molecules that cannot be separated without breaking bonds. As previously discussed, such non-covalent structures can be challenging to conventionally characterise, whilst ion mobility-mass spectrometry is uniquely suited to analyse this class of supramolecules.

**FIGURE 12:**
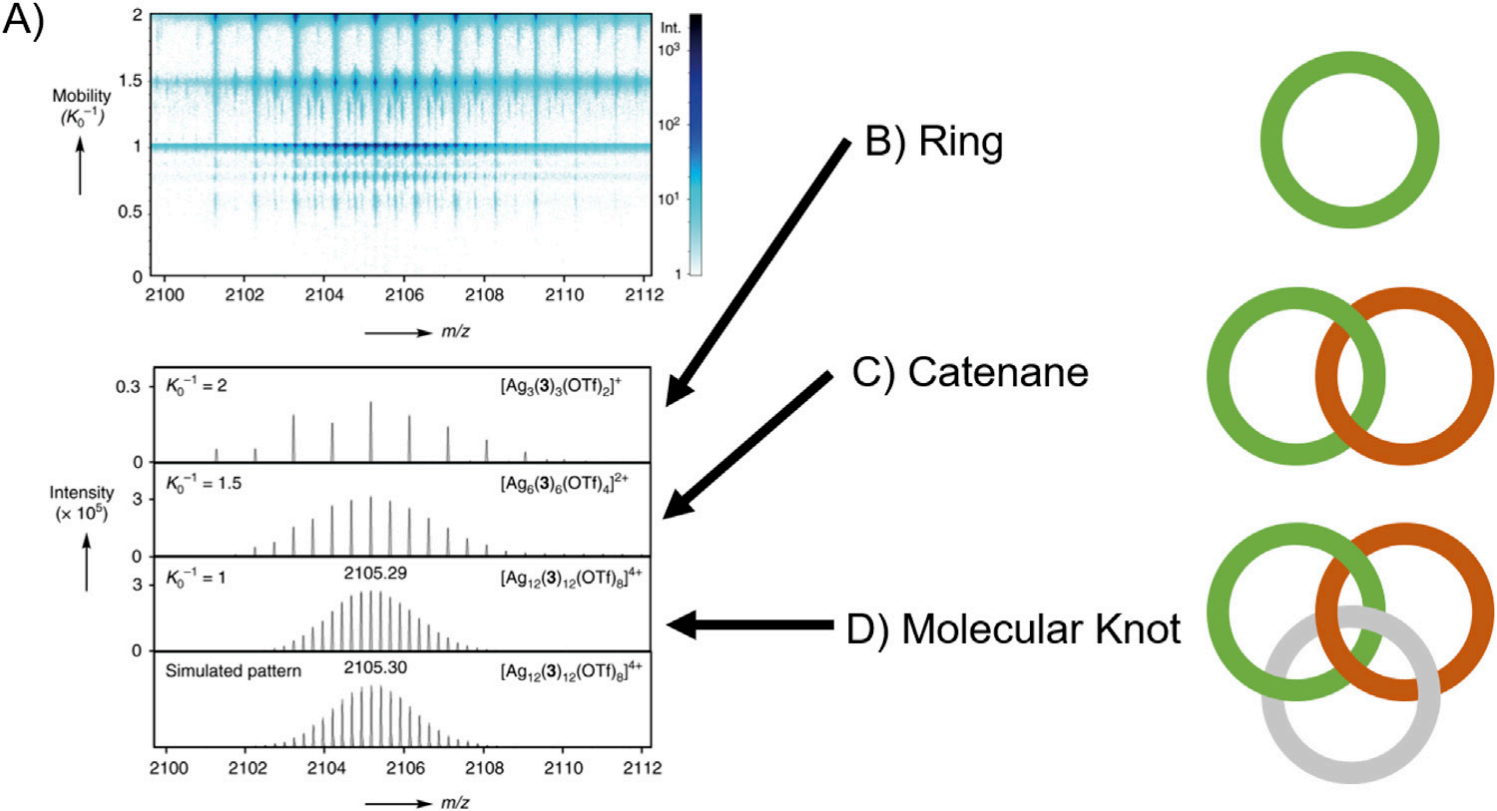
The general concepts that separate **(B)** rings, **(C)** catenanes and **(D)** molecular knots is the number of rings and number of ring crossings. The separation of isobars **(A)** is a simple but effective use of IMS-MS to study catenanes and knots. Here the separation of a complex molecular knot is distinguished from its component rings and catenanes also present in the reaction (Sawada et al., 2019).

Catenanes and molecular knot structures are structures where ring assemblies are interlinked without being chemically bonded (Figures 12A–C). The characterization of trefoil knots formed by active-metal-template knotting reaction by Barran et al. is a first example utilising IMS-MS to analyse this class of structure (Barran et al., 2011). While an elegant Cu^I^ catalyzed click reaction was used to create crossing points of the knot, an equally elegant DTIMS-MS characterisation was employed, in combination with NMR, to differentiate building block and products.

More recently, Sawada et al. synthesised silver-peptide coordination molecular knots (Sawada et al., 2019). Analysis of these was performed using TIMS-TOF, which separated and identified several isobaric catenate intermediates and the product molecular knot (Figure 12). This is an elegant example of the separation of obfuscated intermediates, shedding light on the mechanism by which sophisticated topology is achieved. Investigation of experimental collision cross sections might have further assisted in identification of the species present.

One interesting challenge analysing catenanes is distinguishing between different numbers of crossings for the same number of rings. Using TWIMS-MS catenanes, trefoil knots and other structures were distinguished from each other (Kruve et al., 2019). A “floppiness” factor that compared the arrival time of more complicated knot precursor structures to the least entangled fragment was employed to distinguish the number of crossings. This “floppiness” factor is ripe to be applied to molecular knots formed via CDSA. There is a clear opportunity for probing the topology of complex, interlinked structures, using IMS-MS. CDSA has been shown to create ideal targets for these investigations (Singh et al., 2020).

Rotaxanes are complexes consisting of a wheel and an axle; TWIMS was recently used to assess the structure of a three “wheel” rotaxane (Hanozin et al., 2017). Whilst the driving process for the initial assembly of the oligorotaxanes was not coordination, coordinating counterions directly influenced the secondary structures observed. Increasing the assembly charge in the absence of stabilising counterions, the coulombic interactions between the charged wheels became increasingly repulsive. This led to a conversion from globular to elongated “foldamers,” observed via a marked change in Ω. One interesting observation from the calculated Ω values was an initial shrinking of the globular conformation with an increase in charge state. This was assigned as the result of the decreasing number of counterions incorporated within the structure, although this was not observed experimentally. The coordination of hexafluorophosphate counterions is the driving force for the folding and unfolding of the supramolecule. This is a similar example to the previously discussed charge and counterion induced topological changes, but on a “tertiary” structural level (Mallis et al., 2019; He et al., 2020). Furthermore, two separate instrumental methodologies for switching between the two structures were explored, demonstrating the suitability of IMS-MS for probing the properties of complex structures such as oligorotaxanes. These techniques were electron transfer and collision-induced unfolding, probing the conformational changes induced by charge and internal energy, respectively. In this way IMS-MS successfully unpicked the relationship between counterion and structure.

### Interrogation of the Structure and Function of Complicated Systems

The intermolecular bonds that define supramolecular chemistry can be exploited to impart a wider range of flexibility and “programmable” control than standard covalent linkages could. However, these complicated behaviours are only of value if they are understood. IMS-MS can be a powerful tool for developing that understanding, as these behaviours often yield mass or shape differences (Nortcliffe et al., 2015; Kiesilä et al., 2017; Schröder et al., 2017; Kiesilä et al., 2019).

Self-sorting, one of these exploitable behaviours, is a property of a multicomponent system that exhibits high fidelity molecular recognition between molecules. There are many subsets that define the different modalities of self-sorting, the highest level of which are narcissistic and social. Narcissistic self-sorting involves the self-sorting of one molecule to form supramolecular structures without incorporating any other, whilst social self-sorting involves multiple molecules self-sorting to form supramolecular structures (Safont-Sempere et al., 2011). If no self-sorting is present then the products formed will be a statistical mixture of all combinations available, with all possible results of the self-assembly process as seen in the earlier “snowflakes” (Zhang et al., 2017).

Social self-sorting is a much-desired feature of CDSA systems as this allows the controlled synthesis of targeted heteroleptic supramolecules. Heteroleptic supramolecules are sought after for the ability to expand the range of functionality a single supramolecule can have. Homoleptic supramolecules can have functionality defined by only a single ligand, but can be easier to analyse thanks to an increased level of symmetry (Bloch and Clever, 2017). There are currently competing definitions of self-sorting in CDSA. Any two species that coordinate will be different and so can be considered social. This is commonly overlooked in extending the self-sorting description to the behaviour of ligands, the metals being considered as the bonding areas of ligands, not individual components.

Self-sorting is an intrinsically difficult behaviour to interrogate, more so than self-assembly alone, because all the potential supramolecular assemblies will be formed from the same source molecules, leading to similar sets of signals. IMS-MS is again uniquely situated to differentiate between self-sorted complexes that may otherwise appear similar by other techniques (Liang et al., 2015; Wang et al., 2020c). For example, TIMS-MS succeeded in differentiating between heteroleptic cages where DOSY NMR could not, however these cages were statistical products, not self-sorted (Ebbert et al., 2019).

It was demonstrated in 2010 that FTICR-MS coupled with a mixed flow microreactor could be elegantly used to observe the social self-sorting behaviour of pseudorotaxanes (Jiang et al., 2010). This study is exciting because it highlights how current self-sorting investigations can use IMS-MS to enhance the already rich data from MS. This study successfully identified the intermediates of the main and side product pathways of the self-assembly process, examining structural error correction timescales using only MS. The isobaric rotaxane intermediates could not be separated by MS, but could potentially be separated by IMS. Additionally, self-assembled intermediates could have been interrogated by IMS to reveal structural changes which could have shed light on the mechanism.

Other recent investigations into self-sorting have utilised IMS-MS to varying degrees. Some, such as investigations of ligand spacer length effects on product heteroleptic macrocycles and cages, use IMS-MS to show the clear separation by both IMS and MS of the sorted assemblies (Wang et al., 2018c; He et al., 2020). In these studies IMS-MS is effective because the spacer length change creates clear structural and *m/z* differences that can be identified. Similar earlier work observed narcissistic self-sorting homoleptic cage formation based on the angles of the π-linker structure of the ligands (Wang et al., 2014b). These structure-activity relationships are important to developing novel CDSA systems and IMS-MS provides a clear image of the self-sorting behaviour.

In some instances, self-sorting is not the desired behaviour and can inhibit desired supramolecular structures. Narcissistic self-sorting of individual ligand types will stop social self-sorting of larger heteroleptic cages. One example of this is seen in the work reporting the synthesis of supramolecular hexagrams and pentagrams, where one ligand socially self-sorted to form smaller triangles (Jiang et al., 2017). TWIMS-MS was used to confirm the removal of the triangle from the purified, iron-based supramolecular product. This could be seen in the clean heatmap IMS-MS data presented, showing the familiar step change pattern discussed previously. The cadmium-based analogue could not be separated from the self-sorted triangle by conventional chromatography due to the weaker coordination of cadmium to the ligands. As an extension, potentially IMS-MS could find the Ω of the cadmium product. This value could then be compared to the obtained iron-based data and inform the expected shape and characteristics, despite the inability to isolate it conventionally.

**FIGURE 13:**
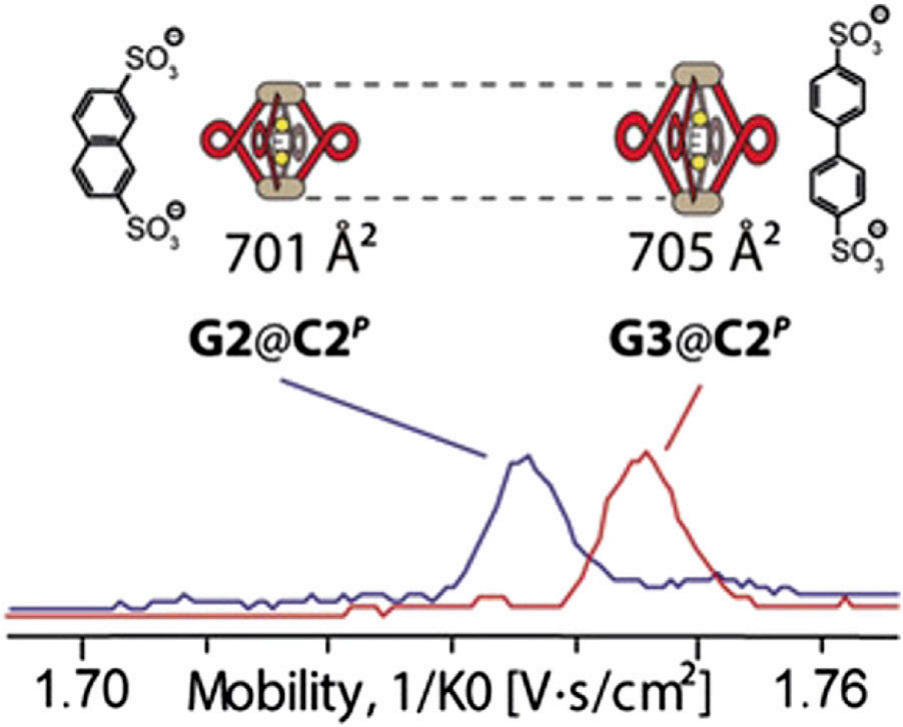
The difference in ion mobility measured by TIMS-TOF of two host-guest complexes can be linked to the change in helical pitch of a constituent ligand as the length of the guest is varied (Schulte et al., 2019).

A behaviour of note is host-guest relationships, a particularly pertinent property in biological systems where enzymes and their substrates are often considered host-guest pairs. Host-guest relationships are a mainstay of supramolecular chemistry defined as “the formation of unique structural complexes between two or more molecules or ions *via* non-covalent interactions.” It could be reasonably argued that many metal-ligand relationships could be characterised as host-guest relationships, akin to the observed behaviour of crown ethers. However, a narrower view of host-guest relationships that limits discussion to larger, supramolecular scales is of more interest, with many supramolecular coordination cages being used as hosts for small molecule guests, with applications in catalysis and biology (Chakrabarty et al., 2011). A reported example of this phenomenon being examined by IMS-MS can be seen in the work investigating the self-assembly of chiral helicene coordination cages. These cages were observed to accommodate two similar aromatic sulfonates, which primarily varied in length. When incorporated into the host guest complex and observed by TIMS-MS, these two host-guest complexes had different mobilities, which could be assigned to a change in the helical pitch of the helicenes within the cage to accommodate the longer guest molecule (Figure 13) (Schulte et al., 2019). This work was used to confirm results obtained via circular dichroism, a technique suited to observation of chiral systems. It is surprising that this work only reported one of the two enantiomers of the cages. It could be interesting to compare mobility results for both enantiomeric complexes.

## Discussion

IMS-MS is now a well-established technique for the analysis of CDSA systems. This review has detailed the manners in which CDSA yields a wide variety of morphologies including 2D cycles and 3D cages, along with opportunities. The versatility of IMS-MS is apparent in the breadth of self-assembled systems to which it has been applied. It can simultaneously act as both a separative and structural technique, even for complex structures and behaviours. This guarantees that its relevance will not diminish as the field of CDSA continues to expand. IMS-MS applications in other fields will inspire similar work within CDSA. Some examples include identifying homologous series using Mason-Schamp based equations (Ahmed et al., 2011) and enantiomer identification (Domalain et al., 2014).

Many of the works discussed here use terpyridine-based binding sites, most notably the tridentate 2,2′:6′,2″-terpyridine (tpy) structural motif (Wang et al., 2020b). Whilst this type of ligand is an effective choice that has clearly led to many derived structures, it also represents only one possible binding site within many reported. IMS-MS is not limited or restricted to a particular ligand binding motif and ESI can preserve even very weak coordination bonds, thus it would be of benefit to study a broader range of ligands to develop an even stronger IMS-MS methodology applied to CDSA. For example, it was seen in this review that the charge of ion will impact the shape and tpy is a neutrally charged binding site. Comparison with negatively charged binding sites could give more information into the role of electrostatic interactions in deciding structure.

As pointed out by Gabelica et al. (2019), reporting of IMS-MS data remains inconsistent and requires clarity. Some of the papers reviewed here did not provide adequate information for experiments to be replicated effectively or for results to be meaningfully compared with other work. This is exacerbated by the dominance of TWIMS, where comparable results are dependent on the calibration used. Gabelica et al. suggest an urgent priority for the IMS-MS community is to decide on a set of calibrant materials and their values (Ω, *K*) and liken the state of IMS-MS to the state of MS prior to the definition of C-12. Perhaps a well-designed self-sorting CDSA system could prove to be a suitable calibrant, which could provide a complex mixture as a calibrant solution containing small molecules, metals and supramolecules. This would give the ability to calibrate instruments across a large range of *m/z* and Ω values with a single solution.

As it stands, IMS-MS can yield complimentary results to well-established techniques such as NMR and crystallography, whilst also being easy to use. More researchers in the field of CDSA should consider augmenting their analytical workflow with IMS-MS to take advantage of the rich data it produces.
